# On the role of the terminal oxidase cytochrome *bd* in hyper-resistance of *Listeria monocytogenes* to the macrodiolide antibiotic tartrolon B

**DOI:** 10.1371/journal.pgen.1011803

**Published:** 2025-08-26

**Authors:** Vitaliy B. Borisov, Elena Forte

**Affiliations:** 1 Belozersky Institute of Physico-Chemical Biology, Lomonosov Moscow State University, Moscow, Russia; 2 Faculty of Bioengineering and Bioinformatics, Lomonosov Moscow State University, Moscow, Russia; 3 Department of Biochemical Sciences, Sapienza University of Rome, Rome, Italy; Texas A&M University, UNITED STATES OF AMERICA

Our attention has been drawn to a recent report by Engelgeh and coworkers in *PLOS Genetics* [1] on the resistance of *Listeria monocytogenes* to the macrodiolide antibiotic tartrolon B. *L. monocytogenes* is the causative agent of listeriosis, a life-threatening infection in newborns, immunocompromised individuals, elderly individuals, and pregnant women. The bacterium developed different resistance mechanisms to antimicrobial compounds produced by nearby competitors. Tartrolon B is one of such compounds. Being a potassium ionophore, tartrolon B dissipates the membrane potential without pore formation [2]. Presumably, the compound not only transports K^+^ out of the cell but can also transport H^+^ into the cell, thereby acting as a coupled protonophore/ionophore [1]. This would dissipate both components of the proton motive force (PMF), electrical (transmembrane electric potential difference) and chemical (transmembrane pH difference). Previously it was shown that TimAB, an ABC-type drug transporter, confers resistance of *L. monocytogenes* to the compound by excreting it from the cell [2]. Now Engelgeh et al. have discovered a second mechanism of bacterial resistance to tartrolon B [1]. This involves ClpCP2 and ClpXP2 proteasomes, the redox-responsive transcription factor SpxA1 (that is the ClpP2 substrate) and its protease adaptor YjbH, which jointly control tartrolon B resistance.

Notably, SpxA1 strongly activates transcription of the *cydABCD* operon that encodes the terminal oxidase cytochrome *bd* [1,3]. Consistently, transposon mutants with impaired cytochrome *bd* function were depleted from a *L. monocytogenes* transposon mutant library following tartrolon B exposure according to Tn-Seq [1]. Thus, the function of cytochrome *bd* is specifically needed for the compound resistance.

The aerobic respiratory chain of *L. monocytogenes* contains two different terminal oxidases, cytochrome *bd* and cytochrome *aa*_3_ [4]. The latter is encoded by the *qoxABCD* operon. Both enzymes use menaquinol as the electron donor to reduce O_2_ to 2H_2_O, and this exergonic reaction is coupled to the PMF generation. Cytochromes *bd* and *aa*_3_ belong to two structurally and evolutionarily unrelated superfamilies, copper-lacking *bd*-type oxidases and heme-copper oxidases, respectively [5,6].

The question then arises, why is it cytochrome *bd*, but not cytochrome *aa*_3_, that makes *L. monocytogenes* resistant to tartrolon B? What advantages does the *bd* oxidase have over the *aa*_3_ oxidase? The main function of heme-copper oxidases in bacteria is to produce PMF to fuel ATP synthesis by oxidative phosphorylation. Bacterial *bd*-type oxidases, apart from carrying out the bioenergetic function, can provide protection against various stresses. In particular, it was shown, mainly on *Escherichia coli* as the model, that a *bd*-type enzyme enables the microbe to survive adverse conditions induced by hydrogen peroxide, nitric oxide, peroxynitrite, sulfide, carbon monoxide, cyanide, ammonia, and some antibiotics ([7,8] and references therein). For this reason, the authors reasonably hypothesized that the addition of tartrolon B to *L. monocytogenes* would cause peroxide-mediated oxidative stress, from which cytochrome *bd* can protect. However, when they tested in a checkerboard assay for synergism between the compound and H_2_O_2_, no synergistic effects on the growth were observed (S9 Fig in [1]). This led the authors to conclude that tartrolon B does not cause oxidative stress. As only one form of oxidative stress was tested (H_2_O_2_), it might be worth investigating further whether tartrolon B exerts any other stresses that cytochrome *bd* is known to alleviate – including the other forms of oxidative stress not tested (see above). Of course, the *E. coli* observations may not be generalizable to other organisms. However, for instance, it was reported that in *L. monocytogenes* cytochrome *bd* rather than cytochrome *aa*_3_ confers resistance to reactive nitrogen species [4].

The authors suggested that the mechanism by which the *bd* oxidase endows resistance to tartrolon B in *L. monocytogenes* is to counteract the compound-induced dissipation of PMF [1]. The maintenance of PMF in the cell is known to be essentially needed for ATP production by PMF-driven F_1_·F_o_-ATP synthase or for the active transport of solutes across the membrane. The idea that tartrolon B could directly inhibit the terminal oxidases seems less likely to the authors “since the Δ*cydAB* mutant is sensitive and not resistant to tartrolon B” [1]. But, in our opinion, the authors’ suggestion as such is somewhat inconsistent with their data. The authors studied the effect of *cydAB* and *qoxAB* deletions on the compound resistance of *L. monocytogenes* (Fig 8C in [1]). The MIC assay showed that the resistance of the Δ*cydAB* mutant to tartrolon B decreased by 5 times while the resistance of the Δ*qoxAB* mutant to the compound did not change significantly compared to the wild type. The milder depletion of *qox* mutants observed in the Tn-Seq experiments, in which genes required for tartrolon B resistance were identified (Tables 1 and S2 in [1]), is in agreement with the data of the MIC assay.

However, the bioenergetic efficiency (the proton/electron stoichiometry) of *bd*-type oxidases proved to be one and a half to two times lower than that of heme-copper oxidases ([9] and references therein). In other words, in *L. monocytogenes* cytochrome *bd* must be a worse PMF generator than cytochrome *aa*_3_. This is because the enzymes produce PMF by different mechanisms. The *bd*-type oxidases do it solely by transmembrane charge separation and are unable to pump protons [10]. The heme-copper oxidases on the contrary are true proton pumps [11]. For this reason, the authors would have seen the opposite of what they observed: the Δ*qoxAB* mutant would have been more sensitive to tartrolon B than the Δ*cydAB* mutant.

Nonetheless, we agree with the idea that the terminal oxidase may be needed to maintain PMF in *L. monocytogenes* in the presence of tartrolon B. We hypothesize that the compound can directly interact with cytochrome *aa*_3_ within the membrane, thereby inhibiting its function, but it does not affect the activity of cytochrome *bd*. In this case, the *aa*_3_ oxidase inhibited by tartrolon B would be unable to counteract the dissipation of the proton gradient, but the *bd* oxidase would still be able to do so in the presence of the compound. This would also be consistent with the observation that the Δ*cydAB* mutant is susceptible to tartrolon B [1].

**Fig 1:**
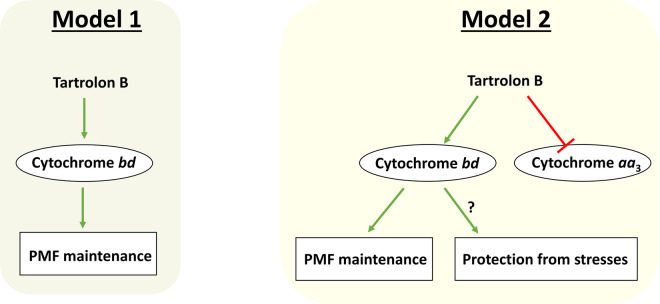
Possible role of terminal quinol oxidases in tartrolon B resistance in *Listeria monocytogenes.* Graphic sketch summarizing two models – the one proposed by Engelgeh et al. [1] (Model 1) and the alternative proposed here by Borisov and Forte (Model 2). According to Model 1, the *cydAB*-encoded cytochrome *bd* endows resistance to tartrolon B in *L. monocytogenes* by counteracting the compound-induced dissipation of proton motive force (PMF). According to Model 2, tartrolon B targets the function of the *qoxABCD*-encoded cytochrome *aa*_3_, directly or indirectly, but does not affect the function of the less bioenergetically efficient cytochrome *bd*. As a result, the unaffected cytochrome *bd* remains the only quinol oxidase capable of maintaining PMF in the presence of tartrolon B and possibly protecting *L. monocytogenes* from stresses induced by the compound.

To clarify the issue, we think that it is important to test in the future whether tartrolon B interacts with and inhibits cytochrome *aa*_3_, whereas it has no effect on cytochrome *bd* in *L. monocytogenes*. The interaction of cytochrome *aa*_3_ with the compound could either inhibit the oxidase activity per se or inhibit only proton translocation coupled to turnover retaining steady-state oxidase activity and making it a non-pumping enzyme [12]. It is also possible that tartrolon B impacts cytochrome *aa*_3_ function indirectly. The two hypotheses, the one proposed by Engelgeh et al. [1] and the alternative we propose here, are summarized in Fig 1.

As a final note, the experiments revealing that the Δ*cydAB* mutant has a fivefold reduction in tartrolon B resistance [1] were not followed up by complementation of the mutant to clearly establish the involvement of *cydAB* in the resistance. Similarly, overexpression of cytochrome *bd* is expected to lead to increased tartrolon B resistance; however, this was not performed by Engelgeh et al. These missing experiments would also be important to further interrogate the possible role of cytochrome *bd* function in tartrolon B resistance.

Deciphering the molecular mechanism by which cytochrome *bd* confers tartrolon B resistance to *L. monocytogenes* will allow us to better understand the mechanisms of antibiotic resistance in other pathogenic bacteria that also involve the *bd* enzyme. This will also help us to develop effective and selective inhibitors of this type of terminal oxidase which could become next-generation antimicrobials.
